# Modelling the Longevity of Dental Restorations by means of a CBR System

**DOI:** 10.1155/2015/540306

**Published:** 2015-03-19

**Authors:** Ignacio J. Aliaga, Vicente Vera, Juan F. De Paz, Alvaro E. García, Mohd Saberi Mohamad

**Affiliations:** ^1^Department of Conservative Dentistry, Complutense University of Madrid, Plaza Ramón y Cajal, s/n, 28040 Madrid, Spain; ^2^Department of Computer Science and Automation, University of Salamanca, Plaza de la Merced, s/n, 37008 Salamanca, Spain; ^3^Artificial Intelligence and Bioinformatics Research Group, Faculty of Computing, Universiti Teknologi Malaysia (UTM), 81310 Skudai, Johor, Malaysia

## Abstract

The lifespan of dental restorations is limited. Longevity depends on the material used and the different characteristics of the dental piece. However, it is not always the case that the best and longest lasting material is used since patients may prefer different treatments according to how noticeable the material is. Over the last 100 years, the most commonly used material has been silver amalgam, which, while very durable, is somewhat aesthetically displeasing. Our study is based on the collection of data from the charts, notes, and radiographic information of restorative treatments performed by Dr. Vera in 1993, the analysis of the information by computer artificial intelligence to determine the most appropriate restoration, and the monitoring of the evolution of the dental restoration. The data will be treated confidentially according to the Organic Law 15/1999 on 13 December on the Protection of Personal Data. This paper also presents a clustering technique capable of identifying the most significant cases with which to instantiate the case-base. In order to classify the cases, a mixture of experts is used which incorporates a Bayesian network and a multilayer perceptron; the combination of both classifiers is performed with a neural network.

## 1. Introduction

**Figure 1:**
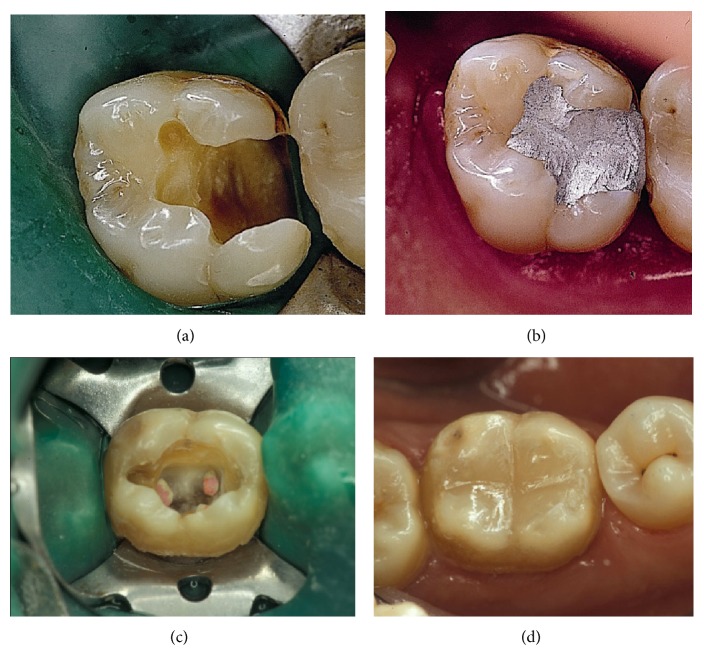
(a) Posterior tooth preparation for amalgam restoration. (b) Posterior tooth restored with amalgam. (c) Posterior tooth preparation for composite restoration. (d) Posterior tooth restored with composite.

The longevity of dental restorations is essentially defined by the material used, although other contributing factors include the characteristics of the cavity, the patient's personal habits, and the dentist's ability [1]. The present paper focuses on the choice of materials and the longevity of placed restorations. An analysis of the reasons for choosing a replacement will be reported in a separate article. The materials that are currently used to restore Class I and Class II cavities are amalgam and composite resin Figure 1. Generally, amalgam is most frequently used, although other materials such as composite are more aesthetically pleasing and have adhesive properties, resulting in reduced preparation size and reinforcement of the remaining dental structure [2]. Composite does not, however, have good results in certain restorations for secondary caries [3, 4]. Studies such as [5] present the factors related to the patient, operator, tooth, cavity size, and materials, although it is not possible to determine the level of relevance as indicated in the study [5]. Long-term studies have shown controversial results regarding the same items: tooth, cavity size, and cavity type. There are reports indicating that composite restorations in Class II cavities, in molars, and in large teeth have a higher potential of failure [6, 7].

Case-based reasoning (CBR) systems have been used successfully in several domains such as diagnosis, prediction, control, and planning [8–11], as applied to different fields. A case can be defined as a past experience and is composed of three elements: a problem description, which describes the initial problem, a solution, and the state achieved when the solution is applied. A CBR manages cases (past experiences) to solve new problems. The way in which cases are managed is known as the CBR cycle and consists of four sequential steps which are recalled every time a problem needs to be solved: retrieve, reuse, revise, and retain. Each of the steps of the CBR life cycle requires a model or method in order to perform its mission. The application of CBR to the field of biomedicine is a relatively new field which has been applied to a great variety of medical problems: in health centres as a control method, for hyperthyroidism, for haemodialysis for patients and/or diabetics, for the diagnosis of patient stress levels, and as a predictive method for the evolution of cancers and leukaemia [12–16]. Dentistry is a new area where CBR can be successfully applied; as such, we did not find articles related to the use of artificial intelligence in the prediction of restorations failure. CBR develops predictive reasoning models, applicable to the failure of resin restorations and/or amalgam obturations of the posterior teeth and to identify contributing factors and causes of the failure.

**Figure 2:**
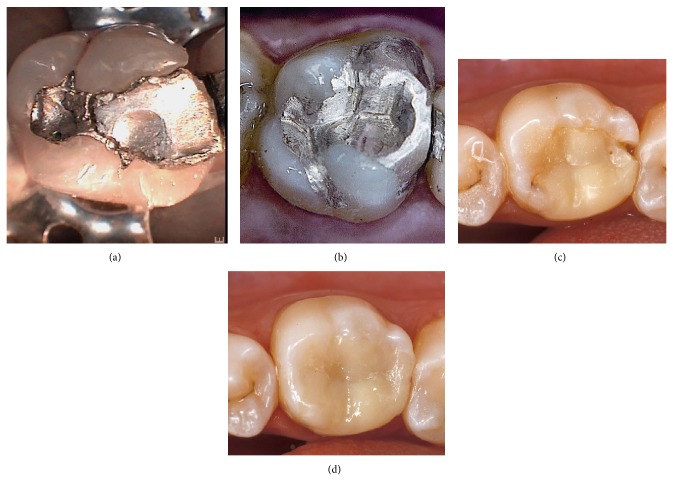
(a) A failed amalgam restoration on a posterior tooth. (b) Posterior tooth amalgam restoration has been redone. (c) A failed composite restoration on a posterior tooth. (d) Posterior tooth composite restoration has been redone.

For this study, a CBR tool was developed to identify the most adequate restoration (composite or amalgam) for a given posterior tooth and for monitoring and predicting the longevity of such restorations.  Figure 2 presents molars which have been treated with amalgam and composite restorations. The retrieval of similar cases is carried out by a clustering process; following the arrival of a new case, the cluster is retrieved by applying the belonging probability according to the estimated parameters for expectation maximization. Once the cases that correspond to a particular cluster have been retrieved, the associated classifiers are also retrieved and recalculated in case they have not been previously trained. An estimate is then made followed by the remaining steps in the reasoning cycle.

This tool guides the practitioners in their work and has proved to be a suitable system for monitoring the state of the art in the field and guiding dentists in the use of different restoration materials. The study of these technological changes could be used to identify the rate of change and the evolution of the complementary restoration techniques: composite and amalgam. This tool was tested with 2,023 patients over the last eight years at the Complutense University of Madrid and the V. Vera Dental Clinic and Surgery Center. The CBR based tool stores records of all patients, including information related to their dental restorations. This information is then used to identify the best possible restoration option and its expected longevity.

This paper is structured as follows.  Section 2 describes the clustering process that was used in this study; Section 3 explains the prediction system; Section 4 details the CBR system; and, finally, Section 5 presents the results and conclusions.

## 2. Clustering

Clustering methods can recover similar information during a given process. In order to apply a clustering technique, it is necessary to take the type of variables into consideration. If there are qualitative variables, it is not possible to compare them directly and it would be necessary to transform the variables from qualitative to quantitative. For this reason, techniques based on neural networks, hierarchy, or partitions cannot be applied to good results, requiring the system to use other alternatives.

In order to manage nominal variables, the system incorporates expectation maximization (EM) [17], which maximizes the probabilities of belonging to each group. Each cluster has a distribution function that differs according to the elements of the cluster. For every distribution function, it is necessary to calculate the required parameters of the distribution function, after which Bayes theorem is applied to calculate the probability of belonging to each cluster.

The process of calculating the parameters is iterative.

(1) (E-Step) Based on the parameters predicted Θ the expectation of the log-likelihood is calculated as follows:
  (1) PV ∣ D,Θt=PU,D,Θt∑D∈DnPU,D,Θt.
(2) (M-step) The new parameters are calculated to maximize the expression according to the expected log-likelihood found on the E-Step. The parameters define the latent variables in the E-Step:
  (2) Θt+1=max⁡Θ⁡PV ∣ D,Θt+log⁡PΘ,

where *U* is the set of data and *V* are the variables.

## 3. Prediction Models

### 3.1. Bayesian Network

Bayesian networks are probabilistic models based on Bayes theorem; they can calculate likelihood according to their constructions. There are several methods to construct a Bayesian network, including the Friedman-Goldsmidtz model [18], tabu search [19], and conditional independence [20], K2 [19], HillClimber [19]. In this case, conditional independence is applied to create the directed acyclic graph (DAG) according to the algorithm of Verma and Pearl [20]. This algorithm creates DAG searching conditional dependencies between pairs of variables *a*, *b*; according to a disjoint set of variables *S*, the condition independence *I*(*a*, *b*∣*S*) is defined by the following expression:

(3) Ia,b ∣ S. If  Pa ∣ S·Pb ∣ S=Pa,b ∣ S,

where *P* is the conditional probability.

### 3.2. Multilayer Perceptron (MLP)

Another prediction method is MLP. The MLP is configured so that the hidden layer has 2*n* + 1 neurons, where *n* is the number of inputs. The MLP also includes bias for each neuron. The activation function is the sigmoidal. According to this configuration, the prediction function is defined according to the following equation:

(4) yjp=fj∑i=1Nwjitxipt+θj.

The inputs are *x*. The inputs and outputs now fall within the range [0.2–0.8] due to the activation functions in the MLP. The value *w*_*ji*_ is the weight, the first index is the neuron in the hidden layer, and the second index is the neuron in the input layer; *θ*_*j*_ is the bias in the hidden neuron *j*, *N* is the number of neurons in the input layer, and *t* is the iteration.

The backpropagation learning algorithm is used [21]. By using the existing definition of the bias and according to the activation functions, the update of the weight and bias in the output and hidden layer is defined according to (5)–(8), respectively, as follows:

(5) wkjpt+1=wkjpt+ηdkp−ykp1−ykpykpyjp+μwkjpt−wkjpt−1.

(6) θkpt+1=θkpt+ηdkp−ykp1−ykpykp+μθkpt−θkpt−1.

(7) wjipt+1=wjipt+η1−yjpyjp×∑k=1M(dkp−ykp)(1−ykp)ykpwkjxip+μwjipt−wjipt−1,

(8) θjpt+1=θjpt+η1−yjpyjp×∑k=1M(dkp−ykp)(1−ykp)ykpwkj+μθjpt−θjpt−1,

where *w*_*kj*_ is the weight, the first index is the neuron in the output layer, and the second index is the neuron in the hidden layer; *t* is the time, *d*_*k*_^*p*^ is value to obtain in the neuron *k*, *y*_*k*_^*p*^ is the value obtained in the output neuron *k*, *y*_*j*_^*p*^ is the value obtained in the hidden neuron *j*, *η* the *μ* the momentum and learning rate, and *θ*_*k*_^*p*^ bias in the output neuron *k*. *M* is the neuron in the hidden layer; the other variables in the hidden layer are defined in similar way to the variables in the output layers.

### 3.3. Mixture

**Figure 3:**
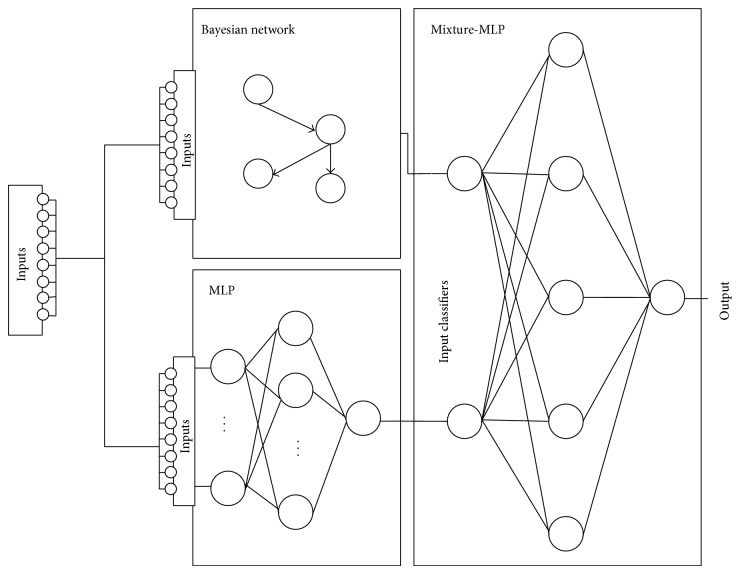
Final estimate based on the output values of the classifiers.

The term* mixture of experts* can be found in works such as [22–24], although this idea can also be found in techniques such as Bagging [25] and Ada-Boosting [26]. The mixture of experts uses a neural network, as in the study carried out by [23], which facilitates the combination of outputs by both methods and reduces error. The neural network selected for this study is the multilayer perceptron.  Figure 3 illustrates the process of mixing based on classifiers. As shown, the output of the classifiers corresponds to the input of the neural network.

## 4. Applying Case-Based Reasoning System to Dental Restoration

The aim of the present retrospective case-based study is to compare long-term clinical performance of amalgam and composite restorations and to identify the best possible treatment.

The technical simplicity and the excellent mechanical behavior of the silver amalgam justify its widespread use in operative dentistry. Since its introduction more than a century ago, composites have unquestionably acquired a prominent place among the filling materials employed in direct techniques. Despite the fact that both amalgam and composite resin are considered suitable materials for restoring cavities, the system presented in this study facilitates the decision-making process for dentists and helps to identify the most adequate material (amalgam or composite) for a given restoration. The developed CBR system also provides information related to the duration of the restoration and can be used to analyze the evolution of both types of materials.

**Figure 4:**
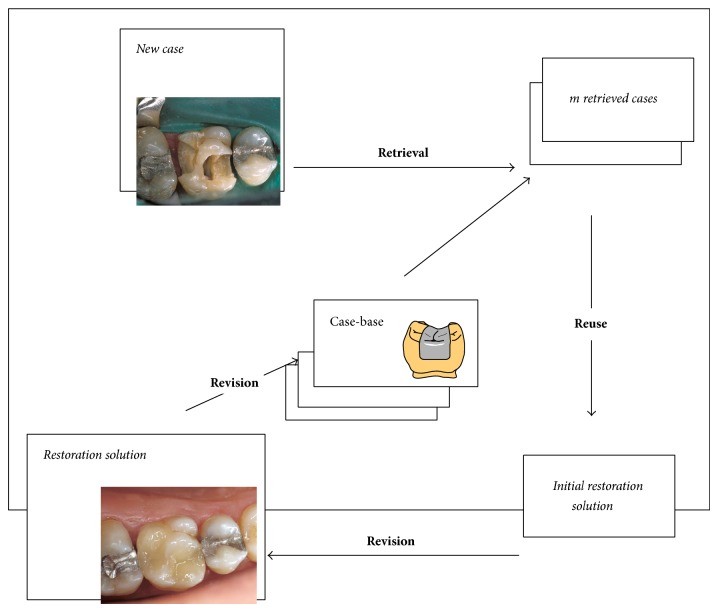
CBR system architecture.

A case-base has been in development since 1993 and now includes information for over 2,000 patients from the Odontology Faculty of the Complutense University and the V. Vera Dental Clinic and Surgery Center of Madrid. These patients were selected because they regularly visit the clinic. A CBR system was selected for its capacity to handle enormous amounts of data, adapt to changes in the environment, and provide an adequate framework for integrating different complementary clustering and classification techniques. The cyclic CBR process proposed is shown in Figure 4.

In Figure 4, the words in bold (shown with arrows) represent the four steps of a typical CBR life cycle, and the words in italics represent data coming in or out of the case-base (situated in the center of the diagram). The case-base used by the developed system was generated using historical records of a number of selected patients from the Odontology Faculty of the University Complutense and the V. Vera Dental Clinic and Surgery Center of Madrid. The present case-base included 4,336 records corresponding to 2,023 patients. Each case includes information for a restoration performed for a given patient. The case-base may, therefore, include several cases corresponding to different restorations of the same patient. This paper presents an improvement of a system initially developed and presented in [27]. In the initial system, a new problem case is constructed when the new patient arrives. This problem case includes information about the patient and the tooth that should be repaired. This new case is used to retrieve *m cases* from a collection of previous cases using Bayesian network and the cluster determined by the EM algorithm. The *m*-retrieved cases are adapted by a mixture of classifiers composed of a Bayesian network and a MLP neural network; the mixture of both classifiers is done using a new MLP neural network. Through the revision process, the proposed solution is adjusted to generate the* final solution* using the confidence limits from the knowledge base.* Learning* (retaining) is achieved by storing the proposed restoration and knowledge (ANNs and the Bayesian network) acquired.

This initial CBR system was successfully tested and used from 2003 to 2011. Improving this system was our challenge and this section will outline the modifications that were made with the intention of demonstrating that the new CBR system can provide successful results and automate the retrieval of cases.  Table 1 shows the changes that were made in the CBR system for evaluating the longevity of dental restorations.

Table 1 outlines the changes made to the original system. The first column of the table indicates the step of the CBR life cycle where the changes were made; the second column indicates the method originally used (and now eliminated); and column three indicates which methods were included in the system. The changes indicated in Table 1 were introduced with the intention of developing a robust model, based on a technology that is easy to implement and that can automate the process of defining the retrieval, reuse, and learning steps of the CBR system. We shall now present the structure of a case and indicate how the kernel methods were used in the three CBR steps described [28].

### 4.1. Case Description

A systematic method was used to research, identify, select, and critically appraise the relevant charts in order to answer the research question in an evidence-based manner.

Each base includes information of both the tooth and the patient. Tables 2 and 3 present the attributes that form part of a case.  Table 2 lists the attributes related to the patients and Table 3 shows the information stored for the teeth of each of the patient treated. There is a case for each restored tooth. Some attributes are Boolean values and others are numerical values, pondered from 1 to 10. The case-base was constructed with 4,336 cases collected from 1993 to 2003, and the system was tested between 2003 and 2011 on 1,714 problem cases.

Dr. Vera saw the subjects annually for follow-up, oral examinations, and bitewing radiographs, at which time they performed complete dental charting, and noted any new treatment needs. We considered restorations needing replacement to be failures. Dr. Vera proposed conducting this study to predict the failure of using composite and amalgam in the posterior teeth and to identify the most important factors. This information will allow clinicians to determine the type of restoration that is best suited for the patient by predicting the longevity of each procedure and using this technique more effectively, thereby achieving higher rates of success in treatments [6, 7, 14].

The CBR identifies the most adequate restoration type and its longevity.

We subsequently classified failures occurring before that point by reviewing the clinical record. We noted several tooth and restoration characteristics to further investigate their relationship with failure [29].

Table 2 contains information related to the last restoration of a given tooth (*restoration date*) and information about the date at which the restoration failed (*restoration duration*). Most restorations do not fail, and in this case the attribute* restoration duration* is the present date. 43 attributes define a given case. The first 41 attributes are the problem descriptor, and attributes 42 and 43 are the solution. The aim of the CBR is then to identify the most adequate restoration type and its longevity.

### 4.2. Creating the Case-Base with Expectation Maximization

We use expectation maximization to organize the case memory. The number of groups is automatically established through a 10-fold cross validation process which calculates the log-likelihood for the validation. The log-likelihood is calculated for each iteration, resulting in a total of 10 measurements for each number that can be statistically analyzed to determine if there is any improvement. The number of final groups is defined by the variation of the maximum log-likelihood during the clustering process. The value is calculated as a product of probabilities from the credibility function. The number of clusters is increased until the value does not exceed 10^−6^. The final number of clusters is limited to 3 with a log-likelihood value of −2.21.

### 4.3. Identification of the Initial Restoration Solution

Several experiments incorporating a mixture of experts have been carried out to illustrate the effectiveness of the CBR system. As previously mentioned, the CBR system has been used at the Complutense University of Madrid and V. Vera Dental Clinic and Surgery Center. The case-base was constructed from 4,336 cases collected between 1993 and 2003, and the system was tested from 2003 to 2011 on 1,938 problem cases.

Table 4 lists the percentage of each type of composite applied between the years of 2003 and 2011. The initial goal was to perform the restoration as predicted by the system, although if the user preferred a different type of restoration process their preferences were respected whenever possible.

In some cases, the system recommendations were not carried out because, for example, the patient was more interested in the aesthetic composite restoration than in the longevity of amalgam restoration. In 12% of the cases, the patients decided to carry out a different type of restoration than the one recommended by the CBR system. On these occasions the percentage of failed restorations was 346% higher than in those cases in which the doctors followed the CBR recommendations. This is clear evidence of the CBR system's effectiveness.

Given the impossibility of empirically validating the long-term results of this model, we will follow up in sixteen years with the amalgam treatment and in eleven years with the composite treatment. We will use the cases stored in the CBR case memory and relate them to the variables of similar cases stored in order to assist Dr. Vera in selecting the most appropriate restorative material for each new case.

## 5. Results 

We have demonstrated a new technique for identifying important cases, which could be used to construct CBR systems. The technique for retrieving cases facilitates the selection of similar cases and the classifiers with which they are associated. The retrieval of the best matching cases is a very simple operation and presents no major computational obstacles. The CBR system allows us to identify the most adequate restorations and provides information about their longevity. The expected longevity of the proposed restoration is obtained by calculating the average tooth longevity from the retrieved cases. As can be seen in Table 5, the average duration of a composite restoration is presently estimated at around 11 years and is 16 years for an amalgam restoration. The developed CBR system was evaluated because it has been operational for 11 years, and on 207 occasions it predicted that the longevity of a given restoration would not last for more than 4 years successfully.

Using restorative treatments with a known period of longevity, we were able to make predictions using a CBR system to estimate the prediction capability of the system. For each of the restorations shown in Table 5, we looked for the most similar restorations and predicted the longevity in order to compare it to the known value. The average absolute error obtained by applying the leave-one-out technique was 0.42 years for a composite restoration and 0.21 for amalgam.

The duration of the treatments can be calculated by analyzing the longevity during the period between 1993 and 1997, according to the duration data obtained up to 2014. This time frame was selected so that the average duration of treatment exceeds the elapsed time.  Table 1 provides a comparison of the values predicted by the initial CBR system, the new proposal, and RBF and MLP without the CBR system. Predictions were compared by applying a 5 × 2 cross validation in which half the data for each year were randomly selected for testing and training, with the process repeated for a total of 5 times. The average estimation errors for each of the methods are shown in Table 6.

The Mann-Whitney test was applied to compare the various methods and determine which was more efficient. H0 establishes that the distribution of both groups is the same, meaning that if we accept H0 there is no significant difference between the methods, whereas H1 indicates that the file method has a lower rank value than the column method.  Table 7 shows the *P* values obtained during the analysis. A value of 0.05 was selected so that if the value is less than this amount, the H0 is rejected.

Table 8 provides information related to restoration failures over the last few years. After eleven years of follow-up visits, we can conclude that the mean annual failure rates of posterior composite restorations were significantly higher than those of amalgam restorations. This was true regardless of the arch, type of tooth, number of restored surfaces, or restoration size. The overall risk of failure due to secondary caries was almost 4 times higher in composite restorations than in amalgam restorations.

## 6. Conclusions

The system allows us to determine the type of restoration that is best suited for the patient by predicting the longevity of each procedure. Using the provided data, the patient can select the type of restoration they prefer. The prediction system used in the mixture was compared statistically with other techniques. We were able to determine that there are statistical differences and that the combination of the prediction techniques with an MLP network makes it possible to select and combine the results of both prediction systems in order to improve the results.

The information which is stored in the CBR system facilitates data analysis and adds to the accumulation of knowledge. The retrieve phase makes it possible to select the most similar cases, grouping the cases with the EM algorithm and using Bayesian network to classify the new case among the available clusters. It automatically generates Tables 4–8, which are of great use to practitioners and can be used for monitoring evolution in the field. Additionally, the system can be used to predict the duration of composite and restoration amalgam.

The system adapts to new cases that it obtains by incorporating new cases to the system case-base and updating the information, which is then used for new predictions. This is an important issue, which can lead to the improvement of results as new cases are introduced in the system.

For future investigation, we have decided to apply the use of the CBR artificial intelligence tool to study and analyse the resin restorations in the posterior area performed by dental students on placement during their studies of Dental Pathology and Therapy II. This will permit us to predict which variables influence the failures, with the aim of correcting the defects of the cognitive knowledge and skills, which have led to the failure of the restorations in the posterior region.

## Figures and Tables

**Table 1:** Changes in the CBR system for evaluating the longevity of dental restorations.

Step	Initial CBR system	Modifications and improvements
Retrieval of cases	*K*-nearest neighbour algorithms	EM algorithm and Bayesian networks

Reuse of cases	Radial basis function network	Mixture of experts

Learning of cases	Radial basis function network pruning metrics	Prediction models and cases

**Table 2:** Case attributes characterising a given patient.

Case attributes number	Attribute	Value
1	Patient number	Real number

2	Sex	Male/female

3	Smoke	Yes/no

4	Drink	Yes/no

5	Date of birth	Date

6	Dental sickness: gingivitis	Yes/no (if yes 1,…,10)

7	Dental sickness: abrasion	Yes/no (if yes 1,…,10)

8	Dental sickness: attrition	Yes/no (if yes 1,…,10)

9	Dental sickness: amelogenesis	Yes/no (if yes 1,…,10)

10	Dental sickness: dentinogenesis	Yes/no (if yes 1,…,10)

11	Dental sickness: pressed dental	Yes/no (if yes 1,…,10)

12	Dental sickness: multicaries	Yes/no (if yes 1,…,10)

13	Dental sickness: other	Yes/no (if yes 1,…,10)

14	Patient sickness: diabetes	Yes/no

15	Patient sickness: high blood pressure	Yes/no

16	Patient sickness: cardiac disease	Yes/no

17	Patient sickness: infectious disease	Yes/no

18	Patient sickness: liver complaint	Yes/no

19	Patient sickness: AIDS	Yes/no

20	Patient sickness: allergic pharmacology	Yes/no

21	Patient sickness: allergic antibiotic	Yes/no

22	Patient sickness: allergic to analgesic	Yes/no

23	Patient sickness: allergic to anaesthetic	Yes/no

24	Patient sickness: allergic to metal	Yes/no

25	Patient sickness: allergic to latex	Yes/no

26	Patient sickness: mental illness	Yes/no

27	Patient sickness: epilepsy	Yes/no

28	Patient sickness: malignant tumors	Yes/no

29	Patient sickness: surgical operation	Yes/no

30	Patient sickness: family history	Yes/no

**Table 3:** Case attributes characterising a given tooth of a patient.

Case attributes number	Attribute	Value
31	Tooth number	1–32
1	Patient number	Real number
32	Restoration date	Date
33	Restoration longevity	Date
34	Restoration type	Amalgam/composite
35	Restoration level	Sample (1–3), composed (4–7), or complex (8–10)
36	Failure fracture of restoration	Yes/no
37	Failure from fracture of dt	Yes/no
38	Failure from accident	Yes/no
39	Failure from caries	Yes/no
40	Aesthetic importance	Yes/no
41	Restoration date	Date
42	**Restoration longevity**	**Date**
43	**Restoration type**	**Amalgam/composite**

**Table 4:** Percentage of amalgam and composite restorations carried out in posterior teeth as suggested by CBR and the dentist from 2003 to 2011 (data from 2011 belong to the first half of the year), at the Odontology Faculty of the Complutense University and of the V. Vera Dental Clinic and Surgery Center of Madrid.

Year	2003	2004	2005	2006	2007	2008	2009	2010	2011

Composite	93%	92%	92%	91%	92%	92%	93%	94%	93%

Amalgam	7%	8%	8%	9%	8%	8%	7%	6%	7%

**Table 5:** Average duration (in years) of amalgam and composite restoration carried out in posterior teeth from 2003 to 2011 (data from 2011 belong to the first half of the year), at the Odontology Faculty of the Complutense University and of the V. Vera Dental Clinic and Surgery Center of Madrid.

Year	2003	2004	2005	2006	2007	2008	2009	2010	2011

Composite	8 years	9 years	9 years	9 years	10 years	10 years	10 years	11 years	11 years

Amalgam	14 years	14 years	14 years	15 years	15 years	15 years	15 years	16 years	16 years

**Table 6:** 5 × 2 cross validation with longevity error in years for the period of 1993–1997.

	Step 1	Step 2	Step 3	Step 4	Step 5
Initial CBR	0.67	0.72	0.82	0.63	0.73

Proposal	0.41	0.54	0.38	0.38	0.45

MLP	0.85	0.95	0.80	1.06	0.79

RBF	1.01	0.84	0.87	1.10	0.81

**Table 7:** *P* values with Mann-Whitney test.

	Initial CBR	Proposal	MLP	RBF
Initial CBR		1.0000	**0.0159**	**0.0079**

Proposal	**0.0040**		**0.0040**	**0.0040**

MLP	0.9921	1.0000		0.2738

RBF	0.9960	1.0000	0.7897	

Bold values indicate a relevant difference, row methods lower than column methods.

**Table 8:** Restoration failure percentage of amalgam and composite restoration carried out in posterior teeth from 2003 to 2011 (data from 2011 belong to the first half of the year), at the Odontology Faculty of the Complutense University and of the V. Vera Dental Clinic and Surgery Center of Madrid. A restoration is considered a failure if it lasts less than 50% of the average duration time for the type of restoration.

Year	2003	2004	2005	2006	2007	2008	2009	2010	2011

Composite	23%	22%	22%	22%	21%	22%	18%	19%	19%

Amalgam	7%	4%	4%	5%	5%	5%	4%	7%	5%
